# A qualitative exploration of women’s experiences of antenatal and intrapartum care: The need for a woman-centred approach in the Peruvian Amazon

**DOI:** 10.1371/journal.pone.0209736

**Published:** 2019-01-07

**Authors:** Harriet Marsland, Graciela Meza, Gilles de Wildt, Laura Jones

**Affiliations:** 1 College of Medical and Dental Sciences, University of Birmingham, Birmingham, United Kingdom; 2 Facultad de Medicina Humana, Universidad Nacional de la Amazonia Peruana, Iquitos, Peru; 3 Institute of Applied Health Research, University of Birmingham, Birmingham, United Kingdom; Monash University, AUSTRALIA

## Abstract

**Objective:**

To explore women’s experiences and perceptions of antenatal and intrapartum care in the Peruvian Amazon, including their perceived motivators, enablers and barriers to accessing care.

**Design:**

Interpretive descriptive qualitative study using semi-structured face-to-face interviews.

**Setting:**

Primary healthcare centre, Iquitos, Peru.

**Participants:**

Women (n = 20) attending the healthcare centre who had given birth in the past 6 months.

**Measures:**

Interviews were conducted using a female interpreter, transcribed clean verbatim and thematically analysed.

**Findings:**

Four core themes relating to antenatal care were interpreted. (1) Perceived knowledge of antenatal care and its importance: women generally understood the importance of care, mainly for their baby’s health rather than their own. (2) Appointments and information received: women wanted more appointments to facilitate greater depth of information relating to their pregnancy. (3) Interaction with healthcare practitioners: women felt they received inadequate attention, care lacked continuity and they were often uncomfortable with male practitioners. (4) Perceived motivators, barriers and enablers to accessing antenatal care: Knowledge of the importance of care acted as the main motivator. Few direct barriers were identified, other than employment. Free care and ease of access enabled attendance. Two core themes were interpreted relating to intrapartum care. (1) Expectations and preferences for labour and delivery: the need for a safe environment for childbirth was acknowledged. (2) Actual experiences of labour and delivery: for most women labour and delivery experiences were not as they had expected. Women objected less to male professionals during labour than antenatal care.

**Conclusions and implications for practice:**

Women reported negative experiences of both antenatal and intrapartum care. There is clearly a need for a more woman-centred approach to care and service provision. Ideally, this would involve employing more staff, acknowledging the implications on resources, improving attitudes towards women, facilitating continuity of care, and allowing patient choice to give women greater involvement.

## Introduction

Antenatal care (ANC) and intrapartum care (IPC) allow for adequate management of the mother and child; minimising complications and optimising health through the early identification of problems and subsequent interventions [1,2]. WHO guidelines [3] focus on a woman-centred approach to ANC, enabling a “positive pregnancy experience” and an “effective transition to positive labour and birth”. They also recommend an increase from a minimum of four ANC consultations, to eight, for all pregnant women globally, with the birth supervised by a skilled attendant.

In Peru, ANC and IPC are delivered by a range of individuals, with the majority of women receiving care from doctors, obstetricians or midwives (Table 1) [4]. In this context a doctor is a trained medical professional specialising in obstetrics, while an obstetrician is a less skilled, non-medical professional trained in the care, diagnosis and treatment of pregnancy and childbirth in normal physiological conditions. A midwife holds a similar role to the obstetrician, but works in the community rather than in healthcare settings. A basic outline of ANC in Peru can be seen in S4 Text. The Ministry of Health have published Clinical Care Guidelines on procedures in obstetrics and perinatal care [5,6]. Despite being an upper middle-income country (UMIC) [7], 98.8% of women receive some ANC in Peru [4], with 95% of pregnant women receiving four ANC visits or more [7]. When compared to other UMICs this is high, with an average of 83.5% of pregnant women in other UMICs meeting this criterion [7,8].

**Table 1:** ANC and IPC Providers.

ANC^†^ Providers	Loreto Region (%)	Peru Total (%)
Doctor	13.8	38.4
Obstetrician	63.9	55.5
Midwife	0	0
Nurse	5.8	3.7
Health Promoter	11.1	1.2
IPC^¶^ Providers	Loreto Region (%)	Peru Total (%)
Doctor	31.0	65.3
Obstetrician	32.6	25.9
Midwife	18.0	3.7
Nurse	1.9	1.3
Health Promoter	2.0	0.4
Family Member	12.0	3.0
None	2.5	0.4

The proportion of different Health Care Practitioners providing ANC^†^ and IPC^¶^ on women in Peru, 2016 [4].

^†^Antenatal Care.

^¶^Intrapartum Care.

The figures showing a high rate of access to ANC might reflect the provision of free ANC by the Peruvian government, through Seguro Integral de Salud (SIS) [9] and the Ministry of Health [10], to pregnant women not covered by other employment or social security insurance schemes. This aims to benefit the most vulnerable and impoverished populations [11].

SIS and other schemes also support the provision of free IPC. However, the Pan American Health Organisation (PAHO) report that the percentage of babies delivered in healthcare institutions in Peru has been found to be below the regional average of 93.8% for Latin America and Caribbean Countries, at 91.4% in 2014 [12]. Furthermore, the maternal mortality ratio (MMR) in 2015 was still high, at 68 deaths per 100,000 live births, when compared to the UMIC average of 41 deaths per 100,000 live births [13]. In April 2018, PAHO and WHO announced their aim by 2021 to achieve a further reduction in this figure to 65 [14]. These data suggest that there is still significant room for improvement in ANC and IPC within the Peruvian setting, with further evidence of significant variations in practice between regions within Peru.

Loreto is the largest and northernmost region of Peru. Statistics show a lower level of access to ANC here when compared to the rest of Peru. 5.4% of Loreto women do not access ANC in their pregnancy, and only 83.5% of women who did access care at any point received that care from a qualified HCP (97.6% Peru total) [4]. Furthermore, the majority of ANC and IPC was delivered by different health care practitioners (HCPs) than in other regions of Peru, with fewer doctors and greater numbers of less skilled professionals attending women (Table 1). This might result in a different standard of care and increased risk for patients in obstetric complications. The region of Loreto had the lowest proportion of births in health care facilities, whilst only 65.5% of births were reported to be attended by skilled birth attendants, compared to an average of 92.4% in Peru as a whole [4]. This figure is less than that found in low income countries such as Zimbabwe and Uganda (78.1% and 74.2% respectively) [15]. Loreto is located in the depths of the Amazon which makes for a unique infrastructure and lifestyle when compared to the rest of Peru. Existing studies suggest underequipped health services and lower effectiveness of health services due to geographical isolation and limited road access to the region [16]. Therefore, experiences and perceptions of ANC and IPC may be specific to this region.

Recent documentation from the Regional Government and Regional Health Directorate of Loreto [17] indicates the need for studies into the quality of care provided to pregnant women, along with the competency of HCPs in providing this.

A quantitative study was recently conducted in the Amazon [18] identifying that women attending ANC in the first trimester were more likely to live with a partner, live in an urban area or have attained education to the level of secondary school or above. A qualitative study [19] explored indigenous Andean women’s experiences of IPC, but detailed none of the findings from the interviews conducted, discussing only the delivery service developed as a result of these. A further qualitative study with this population [20] identified multiple factors associated with accessing or avoiding ANC; including expected criticism for numerous pregnancies, pressure from family members and negative past experiences. The indigenous women had preconceived ideas of mistrust and discomfort with male HCPs, believing that they performed unnecessary examinations. However, diversity in lifestyles between indigenous people residing in the Amazonian rainforest and those in the Andean mountains, along with differences in infrastructure, may result in a difference in culture and service provision.

Consequently, there is a current lack of qualitative evidence from Amazonian women regarding their experiences of ANC and IPC. The aim of this study was therefore to explore women’s experiences and perceptions of ANC and IPC, including their perceived motivators, enablers and barriers to accessing care, in a city in the Peruvian Amazon: Iquitos.

## Materials and methods

### Methodological approach

This paper details an interpretive descriptive qualitative study [21], reported against the *consolidated criteria for reporting qualitative research* (COREQ) guidelines [22] (data in S1 Checklist), allowing an in-depth exploration and understanding of women’s experiences and views surrounding ANC and IPC.

### Setting

Iquitos is the capital city of Loreto, with an estimated population of 471,993 in 2015 [23]. It is a remote and compact city only accessible by plane or boat, attributable to its location in the middle of the Amazon Rainforest [24]. The majority of inhabitants of the city of Iquitos see themselves as indigenous, or descendants of indigenous people [25]. The study was undertaken in a primary healthcare centre in the San Juan Bautista district of Iquitos, serving over 50,000 local people. This was selected as an appropriate site for recruitment, based on its location and provision of ANC and IPC services, with 8 maternity rooms for delivery at the healthcare centre [26].

### Recruitment and sampling

Recruitment occurred during January and February 2017, amongst women attending the healthcare centre with their new-born babies to receive routine vaccinations. Women were eligible to participate if they had given birth in the last six months, were over the age of 18, spoke Spanish and/or English as their first language, and were Peruvian citizens residing in Iquitos at the time of interview. The following exclusion criteria were employed: serious illness or death of the baby, women lacking capacity to consent. Since only a finite window of time was available, convenience sampling was used [27]. Women were first approached in the waiting room by a member of their usual care team, who explained the purpose and nature of the study and screened for eligibility. Those who expressed an interest were then directed to the researcher (HM) and provided with a participant information sheet (data in S1 Text). Recruitment continued until analytical saturation [28] had been achieved and the data collected were deemed sufficiently detailed to address the study’s aim.

### Data collection

Face-to-face semi-structured interviews were selected as an appropriate method of qualitative data collection as they allowed for in-depth exploration of participant’s views [29]. Prior to each interview a demographic questionnaire (data in S2 Text) conducted verbally, facilitating reporting of sample characteristics. A semi-structured interview guide was used (data in S3 Text), based on results of previous studies [3, 19] and discussions between authors. The discussion guide provided some consistency in interviews, but also flexibility in identifying topic areas not initially set out [30]. The topic guide was developed iteratively [31].

All interviews were conducted by HM. Cultural differences were noted between the interviewer, a female University student from the UK, and the participants. A female interpreter was recruited from the Universidad Nacional de la Amazonia Peruana by a local clinician, to account for previous findings that women were less comfortable when the care provider was male [20]. Neither the interviewer, nor interpreter, were involved in the care of the participants. The interpreter signed a statement of confidentiality and was trained to interpret the content of the participant’s words as precisely as possible, ensuring accuracy [32]. All interviews were audio-recorded.

### Data analysis

The interpreter transcribed the two richest interviews in Spanish and translated these into English, to assess accuracy of translation during interviews [32]. Given that few inaccuracies were identified, only the English was transcribed for the remaining 18 audio-recordings. These were transcribed clean verbatim (by HM) to allow for narrative flow [33]. Inductive thematic analysis was used, following the methods described by Braun and Clarke [34]. After 18 interviews, and following an initial review of the transcripts, the interviewer recognised that the narratives were very similar between participants. This was discussed within the research team and two further interviews were conducted before it was decided that analytical saturation had been achieved. Recruitment and data collection was then stopped. Following completion of all interviews, transcripts were repeatedly read to allow data familiarisation and immersion. Two parent themes of ANC and IPC were identified. Preliminary codes were established, based on identified similarities or differences in women’s experiences and opinions, and a codebook created. Codes were then arranged into themes within the two parent themes, discussed with the wider research team, refined and named (HM, LJ). Constant comparison [35] was used throughout analysis, allowing themes to be refined and revised.

### Ethical statement

Ethical approval was granted by the University of Birmingham Internal Ethics Review Committee and the Regional Directorate of Health Loreto. Written informed consent was gained prior to each interview, following the provision of written and oral information.

## Results

A total of 20 women were interviewed. Interviews lasted 24 minutes on average (ranging from 17–36 minutes). Participant demographics have been summarised in Table 2.

**Table 2:** Sociodemographic characteristics of participants.

Characteristic	Number of Participants (%)
**Age**	
18–24	12 (60)
25–29	6 (30)
30–35	1 (5)
>35	1 (5)
**Marital Status**	
Cohabiting *	18 (90)
Single	2 (10)
**Education Completed**	
Primary	5 (25)
Secondary	10 (50)
Apprenticeship	3 (15)
University	2 (10)
**Parity**	
1	10 (50)
2	4 (20)
3	3 (15)
4	1 (5)
5	2 (10)

*Cohabiting: live together and have a sexual relationship without being married [36]

**Fig 1:**
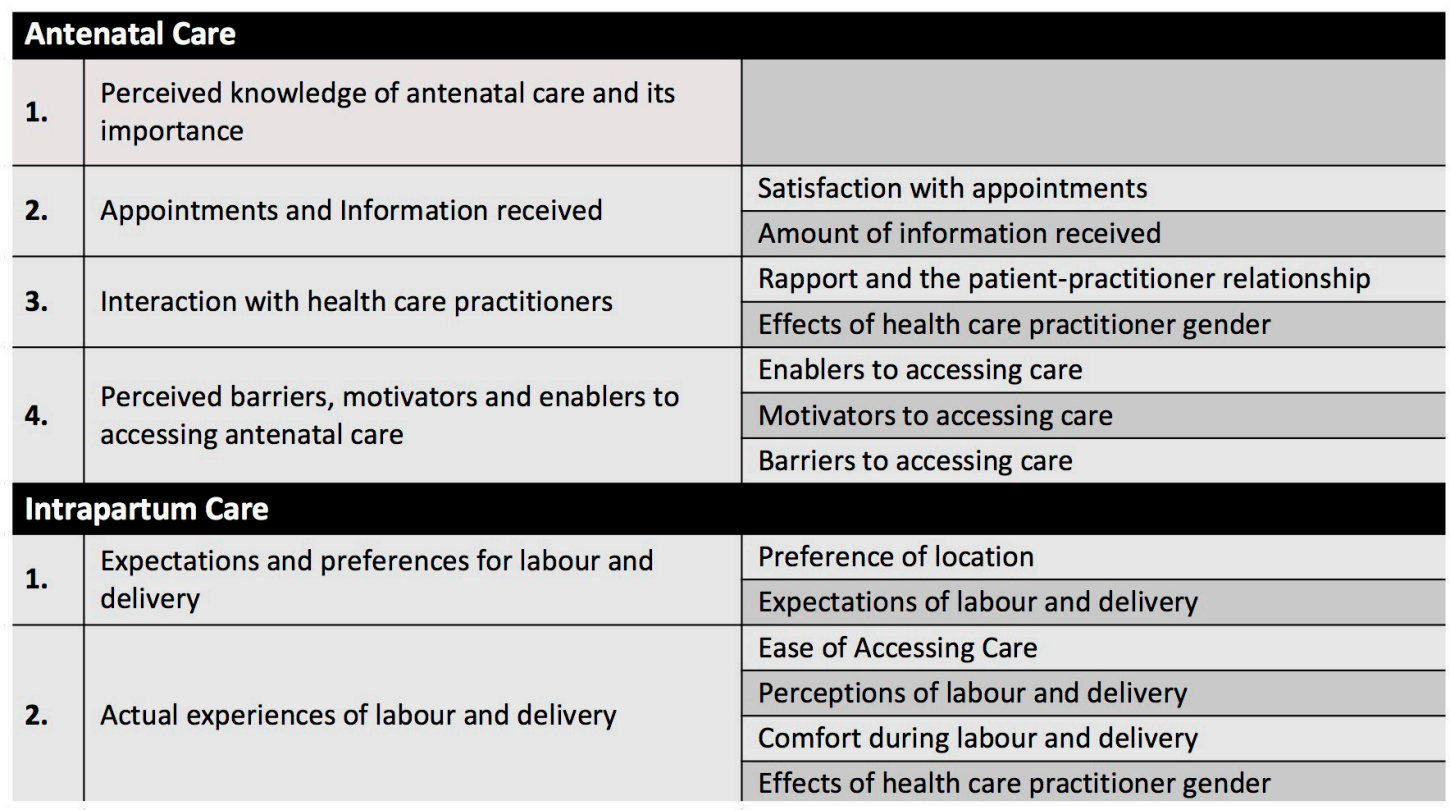
Interpreted themes. The core themes and sub themes interpreted within the narratives around ANC and IPC.

Four core themes and seven sub-themes were interpreted within the narratives around ANC, while two core themes and five sub-themes were interpreted within the narratives around IPC. (Fig 1)

### Antenatal care

#### Perceived knowledge of ANC and its importance

All participants were aware of the importance of ANC, but the perceived level of importance varied. Women recognised that it was a *“human being inside”* (P11) them and care was necessary to *“prevent any dangers”* (P13). Some participants had limited knowledge of why ANC was important: *“I don’t know what would happen to the baby if I didn’t come to the appointments”* (P6) Some referred only to basic functions of appointments, such as monitoring the growth of the baby and the *“day the baby is due”* (P7), rather than the identification of complications. In contrast, others had a better understanding and were able to provide examples of what may happen if they did not attend appointments: *“for example abortion*, *prematurity”* (P3).

The majority of primiparous women identified that it was especially important for them to gain knowledge from ANC: *“because I am a mother for the first time I didn’t have enough knowledge*, *or no knowledge”* of pregnancy (P12). Another said that, because she was *“very young and the baby was [her] first”* (P8), she knew it was more important to engage in ANC. One woman explained that she only understood the need to attend through information provided *“at the beginning I didn’t think that it was so important*, *but when I had the antenatal classes the obstetricians said that it’s very important to have the appointments”* (P19). Multiparous women did not think that ANC was any less important than primiparous women. All participants, regardless of parity, expressed a desire to gain knowledge, and wanted to receive “*more appointments to have more knowledge of the care of the baby”* (P5). Few women acknowledged that the ANC was for both themselves and the baby, referring only to the health of the baby: *“If I didn’t have these appointments the baby could have illnesses”* (P3).

#### Appointments and information received

Satisfaction with appointments:

Several women explained that they *“would have liked to receive more appointments”* (P5). Participants usually received one appointment per month, followed by weekly appointments after eight months’ gestation. Based on this schedule, most women would only have been able to attend more if they had presented earlier or had not experienced barriers, such as employment. One participant, although wanting more appointments, *“agreed with the number of appointments”* (P12) received, acknowledging that you may only receive limited appointments through the clinics. Another participant *“only wanted to receive good care”* (P3), giving greater importance to the quality of the care and information received during appointments, rather than quantity.

Amount of information received:

The women wanted to *“have more knowledge”* to allow for *“thorough care of the baby”* (P7). Most interviewees reported receiving *“a lot of information”*, but there were still things that they thought important to know. For example, *“the sex of the baby”* (P2), *“more knowledge about the development of the baby”* (P9) and *“to teach me how to give birth*, *how to look after myself…what is good and bad for the pregnancy”* (P15). Some also wanted to have *“more scans”* (P17). In antenatal classes, women learnt *“how to care for the pregnancy”* (P19). Without this, one woman *“didn’t know how to have the baby”* (P16). Others believed it to be the best aspect of their care and *“started to want to have the baby”* (P20).

Women reported a lack of consistency in the provision of information. During the antenatal period, some participants reported they *“sometimes felt that [they] didn’t receive enough information”* (P17), especially if seen by impatient HCPs who *“just wanted to see [them] quickly without answering any questions [they] asked”* (P19). Some believed that the *“nurses…didn’t want to give enough information”* (P6). One participant reflected that *“I didn’t know why [they] didn’t give me enough information*. *I don’t know if I had to ask for the information”* (P15). Similarly, there appeared to be a perceived lack of consistency between pregnancies: *“The information for each baby was different”* (P10). The same participant reflected *“that each woman receives different information according to their ideas and beliefs”* (P10). An HCP might have provided different information to women of different cultural backgrounds attending appointments at the same healthcare facility. In contrast however, some participants felt that overall *“the staff who worked here*, *they gave me enough information*, *they explained things very well”* (P16).

#### Interaction with HCPs

Rapport and the patient-practitioner relationship:

Throughout interviews, women appeared to make no distinction between the words obstetrician, doctor and nurse. This might have been a lack of awareness of the differing roles of professionals within the team, simply viewing all members of staff as caregivers.

All women reported receiving appointments from obstetricians throughout their ANC. Of the women interviewed, the majority received their ANC in local primary healthcare facilities through their SIS. These women were *“attended by several obstetricians”* (P17) throughout their appointments, resulting in a lack of continuity in their care. The remainder of participants received care from only one HCP, attending a different healthcare facility such as a private hospital funded via other social security schemes. They commented on the quality of care experienced in these facilities: *“the person who cared for me was very patient and had a great responsibility”* (P16). Women reported hurried appointments with obstetricians who *“just wanted to see [them] very quickly”* (P19). One participant recalled unpleasant experiences when *“doctors are sometimes not kind*, *they’re a little…rough”* (P5). It was reported that *“it was good to feel that the obstetricians were interested in the care of the baby”* (P14). One participant reflected that poor experiences of care were as a result of the patients themselves and not the staff: *“Some people say they aren’t attended with patience here*. *I think that these people are also not patient people…sometimes they have a bad attitude towards the staff who work here”* (P17).

Effects of HCP Gender:

Most of the participants commented that they would *“feel strange and uncomfortable”* (P20) if they were seen by a male HCP, regardless of whether they had experienced this. One woman justified this, saying *“I didn’t like it when the male obstetricians touched my stomach*, *or when they had to look at my parts”* (P15). Another *“received bad appointments from the male nurses”* and believed *“the man…wasn’t patient”* (P2). Many participants expressed the preference for a female HCP: *“I wanted to receive the appointments from a female”* (P2). This view was not universal however; with some reporting that they *“felt good about care from males and females”* (P13).

#### Perceived barriers, motivators and enablers to accessing ANC

Barriers to accessing care:

The majority of participants could identify no direct barriers to accessing care. However, this might be a reflection of the women recruited, since in this study all had attended ANC. Of those able to provide reasons for non-attendance, the main barrier was employment: *“Sometimes I had to ask for a permit from the principal of the school [where I worked] to come here for my appointment…sometimes he didn’t want to give me a permit”* (P12). As previously reported, other women did not have as many appointments as they would have liked because of their work: *“I had to work and didn’t have time to come here for my appointments”* (P14). One working woman attended a hospital where this was not a barrier, since she was able to have her appointments in the evenings (P5). However, the majority of women who worked reported difficulty in arranging appointments at convenient times. A few women referred to the weather as a factor preventing attendance, since downpours and flooding are common in Iquitos: *“sometimes when it rained a lot I couldn’t go to my appointments”* (P7).

Most women reported long waiting times at the healthcare centres, which might have acted as an unconscious barrier to accessing care: *“I had to wait two hours or more”* (P14). Despite care being free, and intended cover of transport costs by the SIS [9], lack of money was still a barrier for some women: *“Sometimes I didn’t have enough money to pay for the transport to get to my appointments”* (P7). Furthermore, some participants felt that having to book their own appointments was awkward and they saw this as a high level of responsibility, which may have acted as a barrier: *“I have a responsibility to book an appointment again”* (P5).

Motivators to accessing care:

All of the women interviewed expressed a motivation to attend appointments: *“I never missed any appointments*. *I believe that the appointments in my pregnancy are very important”* (P20). They reflected that *“a woman who doesn’t visit the doctor or nurse*, *they don’t know how their baby is growing”* (P7). One participant explained that *“I didn’t want to lose my baby by my own responsibility”* (P16). Another interviewee initially had no motivation to attend, but at her antenatal classes *“the obstetricians said that it’s very important to have the appointments and come here for care”* (P19).

Enablers to accessing care:

There were a number of enablers for the women accessing care. Most women had a SIS [9], meaning *“there weren’t any costs”* (P3) when attending appointments. Some women voluntarily paid for further ultrasounds at separate clinics. One participant *“had some problems with [her] SIS”* (P2), so was unable to access ANC until eight months’ gestation: “*they told me that my SIS was not from here and that I had to make a change in order to be served*”. (P2)

The women reported the ease of getting to, and booking, appointments. Most of the healthcare facilities care for *“people who live close”* (P11) and *“it’s very easy to get an appointment”* (P13). The day when the women were required to next attend the clinic was written on their patient-held antenatal card at the end of their consultation: *“the nurse has to give me an exact date to return again”* (P6). Occasionally appointments were booked by the obstetrician, but usually the patient called on the given day, or day before, to arrange the appointment.

### Intrapartum care

#### Expectations and preferences for labour and delivery

Preference of location:

The majority of participants recognised that giving birth in designated healthcare facilities carried fewer risks than homebirths and acknowledged the need for skilled professionals: *“having babies at home is prone to complications and is dangerous”* (P2) and *“they don’t have enough knowledge at homes compared to the nurses and doctors”* (P18).

A couple of women preferred home births: *“For me it’s better to have babies at home because I can be with my family…the family give me good care”* (P6), whereas in facilities any relatives have to wait outside the delivery room. One woman had previously given birth at home with the assistance of her neighbour (P2). Another believed that *“it’s the same to have the baby at home as it is in the hospital*. *The one difference*, *after giving birth to the babies the nurses clean your parts”* (P13). In one instance the main barrier to giving birth at home was the family’s concern for safety: *“my mum didn’t want this baby born at home because she was afraid for me”* (P2). One woman recognised the limitations of deliveries in health centres and that, for this reason, many women were referred to hospitals for caesareans: *“I couldn’t have the baby here because the staff who work here don’t attend to women who are going to have babies by caesarean*. *They don’t have enough instruments”* (P4).

Some women expressed a preference to giving birth in hospitals over healthcare centres following past negative experiences: *“I didn’t want to have more babies there*, *because I felt that I wasn’t cared for very well because the obstetricians weren’t good and patient with me …I decided to have the next baby at the hospital*. *There I felt happier to be cared for”* (P18).

Expectations of labour and delivery:

The majority of women expected a natural birth, without complications: *“I expected to have the baby without problems”* (P15), *“I wanted to have it naturally without caesarean”* (P19). They believed that *“to have babies by caesarean is complicated”* (P7). Women thought they had *“learnt how to give birth to the baby”* (P20) and *“knew all the changes [they were] going to feel”* (P16) during labour.

Women’s expectations of the pain they might experience varied, with multiparous women referring to the pain experienced previously during labour. Of the primiparous women, some believed that they *“wouldn’t feel a lot of pain”* (P4), while another woman commented that *“many people that I met would tell me all the time that I would feel a lot of pain*, *and that I would need to tolerate it”* (P17). Some participants expressed a desire to use pain relief, as they believed it would be effective. The remaining women preferred to *“have babies with all the pain that you get”* (P13), believing that *“nothing would relieve their pain”* (P13).

#### Actual experiences of labour and delivery

Ease of accessing care:

All women interviewed gave birth in either Iquitos Hospital or the healthcare centre. This centre did not have the capacity to conduct caesarean sections. Women made their own way to the healthcare centre upon going into labour, where 24-hour care was available. Since the women were local to the centre, this care was easy to access: “*when I felt the pain I came here immediately to receive the necessary care…I came here by motorbike”* (P6). However, initially some were turned away and told to return later: “*I was already in pain*, *I felt symptoms*. *They made me go back to my house*” (P15). Any women attending the centre for a delivery, who were assessed as needing an emergency caesarean section, were transferred by provided transport to the local hospital: “*I had to have a caesarean at Iquitos Hospital and I was transported there*” (P17). Caesarean sections were planned in only a few of the women, where expected complications of labour had already been elicited and surgical delivery had been deemed to be the safest option. These women made their own way to their local hospital.

In one instance, a woman “*wasn’t seen quickly*” so her mother decided that she “*had to have the baby at Iquitos hospital because there they see you more quickly”* (P19). Some women experienced difficulties when their babies required further attention, providing a barrier to access to care in this period: “*I stayed at iquitos hospital for one week*, *but the baby stayed there for two weeks…I didn’t have enough money to keep coming back home and going to the hospital”* (P17).

Perceptions of labour and delivery:

The majority of women reflected that their experience of labour was not as expected: *“when the doctor told me that I was going to be operated [caesarean section] on*, *I felt afraid because I thought that something could happen to the baby”* (P9). As a result, it appeared that the women were left feeling dissatisfied with their IPC. There was one participant who reported being unaffected by her unexpected method of delivery: “*I thought that it was great…because it was a new experience to have a baby by caesarean”* (P7). Another woman retrospectively agreed, after having to be transferred to Iquitos Hospital for an unplanned caesarean section: “*births are better…at the hospital because I think the staff have enough instruments to help them*”. (P9)

A lot of the women reported that they were unhappy with the care received. They reported that they lacked attention and the nurses were *“so rude with the patients”* (P6), especially prior to birth: *“My waters broke and I lost blood…but the obstetricians didn’t pay attention to me”* (P15). One participant reflected that, while struggling following birth, *“all the nurses were very impatient with me and just told me to breastfeed the baby*, *nothing else”* (P11). Another woman however, reported that it was different after she had given birth compared to during her delivery: *“I felt I got more attention from the nurses…asking if I felt good and didn’t have any problems”* (P14). In healthcare centres specifically, some women felt that they should have been being attended to, but *“the obstetrician was seeing other females”* (P3). Women *“had to wait a long time to be attended”* (P4) and one woman initially attended a healthcare centre for delivery but was taken to hospital by her mother as she *“wasn’t seen quickly”* (P19). One woman reflected that *“the care would be better if staff who worked here…would attend people not just when it is so important and an emergency*.” (P14) Those attended at the hospitals reported being cared for by multiple staff during their labour, sometimes up to *“ten people”* (P1), whereas those delivering at healthcare centres frequently reported the presence of only two or three members of staff.

Comfort during labour and delivery:

Most women reported the area in which they gave birth was clean. However, this was not universal. One woman explained that *“it was dirty”* and in *“the bed where [she] lay*, *[she] felt something bite”* (P6). Another explained that she perceived that her *“cut was infected because [she] had a bad caesarean”* where *“the doctors used dirty instruments”* (P8). Only one woman reported the use of pain relief when giving birth naturally at a healthcare centre (P3). Another woman *“was afraid to ask for pain relief*, *because the nurses sometimes don’t have patience”* (P6). Many women reported feeling *“very afraid”* (P1) and *“anxious”* (P2) during the birth, *“in case there was something wrong with the baby”*. In contrast, some commented that they *“felt relaxed”* (P2) and *“very excited”* (P5). Most women, regardless of their thoughts and feelings at the time, *“just wanted to have the baby here”* (P2).

Effects of HCP Gender:

Largely, women seemed more content receiving care from males during IPC than ANC: *“I didn’t feel afraid [of being attended to by males] because I just wanted to have the baby”* (P5). One participant was still not happy to be attended to by a male but due to the unpredictable nature of delivery this was unavoidable: *“I didn’t feel good*, *I felt I wasn’t comfortable receiving care from the man…I couldn’t do anything because I was going to have a baby”* (P10).

### Similarities and differences between ANC and IPC

Throughout their care, women were more concerned for the health of their baby and not the risks to themselves. They understood the importance of receiving care from skilled professionals. There was a constant desire for information throughout ANC, resulting in many women feeling afraid and unprepared upon commencing IPC, particularly if a caesarean section was necessary. In both areas of care, long waiting times were reported with care delivered by different HCPs and insufficient members of staff. Women reported that they wanted to feel as though they were valued in both ANC and IPC, feeling they lacked attention and were unable to develop a rapport. Due to the nature of the facility women felt they were able to easily access care, with few barriers.

## Discussion

### Principal findings

The knowledge of the importance of ANC for the unborn child motivated women to attend, while the desire to gain further knowledge resulted in a wish for more appointments and better quality of care in order to deliver this. Minimal direct barriers to ANC were identified, which might reflect the fact that the women who participated had accessed care, however employment was perceived to be a problem in the minority of women that worked. Free facilities, ease of attending and booking appointments enabled access to care. A safe environment and a skilled attending physician were recognised as necessary for childbirth. However, women wanted to receive adequate attention in labour, with a desire for increased support from relatives. For some the pain during labour was worse than expected, and women wanting pain relief at the healthcare centre did not receive this. Dissatisfaction with delivery was usually as a result of perceived poor care from obstetricians, or labour not being as the woman had expected.

### Comparison with literature

The patient-practitioner relationship was found to be important in the perceived quality of care received, agreeing with previous findings within the global literature [19,37,38]. Women lacked trust and were unable to establish a rapport because of lack of continuity in the HCPs they saw [19,38]. Studies have also identified negative experiences of care, with women being treated in a brusque manner by HCPs [19,37,39–41]. Poor HCP attitudes have been reported [37,40], with lots of women reported being seen hurriedly and without patience [41], further agreeing with the findings identified in this study that women felt they did not receive sufficient attention.

The wider literature identified that most women felt uncomfortable receiving care from male HCPs during the antenatal period and during labour [20,42–44]. Our study agreed with the finding that provision of ANC by male HCPs made women uncomfortable, however few women were averse to care from males during childbirth. The only study agreeing with the finding that women were less uncomfortable with male HCPs in labour was conducted in Saudi Arabia [45], where women insisted on male surgeons conducting their caesareans. Studies have found that, in some cultures, patients view males as physicians and females as helpers or assistants [46,47].

In this study, women were found to favour births in healthcare facilities. This may be due to the fact that Iquitos, although developing, is a city where clinics are more central and accessible, contrasting with findings in rural Peru where clinics are hard to access and attendance often requires long travel times [48]. Literature from lower income countries suggests that women in rural areas prefer home births, utilising traditional birthing methods [43,48], disagreeing with the current study. This might further reflect the difficulty attending healthcare facilities for women living in rural communities [49].

Previous studies conducted in rural areas, where access to ANC was lower [18], identified direct barriers to accessing care; including long waiting times, mistreatment by HCPs, lack of trust, perceived unavailability of HCPs and transport costs [19,20,37–41,48]. In this study, these only arose as issues through discussions and were not elicited as direct barriers, since the women interviewed had accessed care. Unlike in other developing countries, where care is often self-funded [37,38,40], we found that women benefitted from their SIS and experienced limited financial problems while accessing ANC [9]. Cost of transport was a reported difficulty for some participants; however, the SIS is intended to cover this [48] so this is likely to be an issue of limited concern. Other studies report women preferring to give birth at home due to poor access to facilities [38,44], while those in rural Peru identify a need for motorcycles or ambulances to transport women in labour to healthcare facilities [48]. These were already in place here.

Limited clinic working hours have previously been reported as a problem [38], but employment was uncommon in other studies carried out in developing countries so this has not previously been identified as a barrier [39]. This might be a further reflection of the inner-city location of this study, with more women in employment compared to a rural setting. Many women in Iquitos work in markets, having to close the stall or leave it unattended to attend healthcare appointments [18]. Currently the Peruvian government do not allow employed women to take time off for antenatal visits [50], despite WHO recommendations [51].

Staff shortages within clinics have previously been reported as impacting on ANC and IPC service provision [37–39,52]. This was also alluded to by participants in this study; reporting long waiting times, lack of attention and a hurried manner of appointments. Previously women have frequently reported unsatisfactory, inadequate care and untimely attention in IPC [42,43,52], which has been attributed to poor staff attitudes [39], but could be further attributable to a staff shortage. We found women’s deliveries were not as expected, echoing previous findings that women felt they did not know enough at the time of birth [42,43].

### Strengths and limitations

As far as the authors are aware this is the first interpretive qualitative study to be conducted in a city in the Peruvian Amazon exploring women’s experiences of ANC and IPC. All women included had given birth within the last six months, allowing for a recent account of, and reflection on, their IPC and ANC throughout pregnancy. Participant demographics were varied and reflected different ages, levels of education and parities.

The nature of the recruitment process meant that women attending only one healthcare centre were interviewed, with the majority already demonstrating an interest in their child’s health by attending for routine vaccinations. All participants had engaged in ANC and IPC potentially limiting the identification of barriers to care and views of non-attendees [53].

The interpreter was studying English at University and had little experience of translation, particularly in a research setting. This may have resulted in the misinterpretation of some concepts [32]. However, the interpreter remained consistent throughout all interviews and was trained by the researcher to interpret the interviews. Appropriate mechanisms were in place to reduce the likelihood of misinterpretation, including comparison of transcripts in Spanish and English to assess the accuracy of translation [54]. The interpreter was part of the community and a mother herself so was able to develop a rapport with the participants, allowing the disclosure of more information.

Only one researcher coded transcripts, however analysis and interpretation of data were discussed with the wider research team. The cultural differences between the researcher and participants at the clinic were apparent, but the researcher attempted to remain unbiased throughout interviewing and reflexive during data analysis. The nature of face-to-face interviews meant that participant’s reported experiences might have been subject to social desirability bias [55]. However, the impact of this was thought to be small since all participants appeared to develop a good rapport with the interpreter during interviews.

### Recommendations

As in most publicly funded and social insurance based systems, there are constraints in terms of funding and staff in Iquitos’ free health service, putting limits to service improvements.

Ideally, more staff should be employed, allowing for reduced waiting times and increased attention for patients, with more time for each ANC consultation. More staff at the delivery facility would permit greater attention for each patient, improving the overall delivery experience.

A change in HCP attitudes could improve women’s experiences of ANC and IPC without the need for monetary expenditure. HCPs should show patience, respect and care towards patients. Studies in high income countries suggest increased satisfaction with ANC when all of the care for one patient is delivered by the same HCP [56,57]. Increased personal continuity of care could be beneficial, even if limited by the need for shift patterns of HCPs for routine ANC sessions, and even more so for IPC. This also appears as a recommendation in the most recent WHO ANC guidelines [3]. Women would be able to develop a relationship with their HCP, permitting them to potentially feel more comfortable to ask questions and satisfied with the information received. The option to choose an HCP may empower women, helping to make them feel valued and giving greater involvement in the care received. Furthermore, this could address the problem of discomfort with male HCPs: if the woman had a choice she would be able to actively request a female HCP, or trust could be established through continuity of care with a male HCP. Choice in care could extend to birth, with the women making informed decisions as to whether they wish to give birth at a primary healthcare centre or a hospital.

The WHO recommend [51] that employers allow time off work for pregnant women to attend antenatal visits. Maternity rights in Peru could be modified to enable this, providing adequate antenatal visits for women during pregnancy. For example, in the UK, employers are required to give pregnant women paid time off work to attend all necessary antenatal visits [58]. Alternatively, evening clinics might enable these women to access care at a more convenient time.

Taken as a whole this would enable the implementation of a woman-centred approach to care, providing for a positive experience, as suggested in the WHO guidelines [3]. A woman-centred approach with greater continuity might allow the woman to feel that her health is also a priority, and not just that of the child.

Future studies should investigate the enablers and barriers, not just in those accessing ANC, but also in those only partially accessing care or not accessing care at all. It might also be beneficial for a study to be conducted in multiple healthcare centres to further increase the diversity in demographics of participants interviewed.

## Conclusion

Despite the provision of a free state healthcare system, high attendance at antenatal clinics, ease of access to care and the high rates of healthcare facility births in Peru, women were still dissatisfied with the quality of care received. A woman-centred approach to services and care should be explored helping to provide women with a more positive experience of pregnancy and delivery.

## Supporting information

S1 Text Participant information sheet. (PDF)

S2 Text Demographic questionnaire. (PDF)

S3 Text Interview guide. (PDF)

S4 Text Basic outline of antenatal care. (PDF)

S1 Checklist COREQ checklist. (PDF)
