# Supplementary material for: A qualitative exploration of women’s experiences of antenatal and intrapartum care: The need for a woman-centred approach in the Peruvian Amazon
Source: PLoS One. 2019 Jan 7;14(1):e0209736. doi: 10.1371/journal.pone.0209736 (PMC6322728; doi:10.1371/journal.pone.0209736)
Supplement: S1 Text — (PDF) [file pone.0209736.s001.pdf]

Exploring women's experiences and perceptions of Antenatal and Intrapartum Care in the Peruvian Amazon: a qualitative study

My name is Harriet Marsland, and I am a medical student from the University of Birmingham, England. I am currently at the health centre in Iquitos to conduct a study and I am looking for volunteers to take part in this study. The information collected will help to improve the services provided during pregnancy and childbirth.

I would like to invite you to take part in this research study looking at pregnancy and childbirth care services in Iquitos. This information sheet has been provided to help you decide whether to take part. It is important that you fully understand what this study involves and what will be asked of you if you agree to take part. Please carefully read this information sheet. You will be able to ask the researchers any questions via an interpreter.

Why is this study being conducted?

The aim of the study is to explore the experiences of women receiving care during their pregnancy (known as antenatal care) and the birth of their child (known as intrapartum care). It will also look at their perceptions of the care received and what women think works and doesn't work in antenatal and intrapartum care.

What will the study involve?

Participation in the study will involve filling out a questionnaire. You will then be asked to take part in an informal discussion (known as an interview) with an interpreter, lasting 30-60 minutes. The researcher will talk to you about the care you have received during your pregnancy and when you delivered your baby. The researcher will ask you some personal questions and explore further your understanding of care during pregnancy, and the reasons behind any choices you have made. There are no right or wrong answers to these questions. With your permission, the interview will be audio recorded. The information you provide will be used alongside other interviews to write up results from the study. We may include some quotations from your interview, but nobody will be able to identify you (for example your name will not be used).
If you choose to take part, the interview can be done today. If it is more convenient for you, it can also be carried out at a later date.

Who is needed for this study?

The study is looking for women living in Iquitos who have given birth in the last 6 months. The women who take part need to be willing to talk about the care they received during their pregnancy and birth.

Do I have to take part?

You do not have to take part in this study, participation is voluntary. If you would like to take part, you will be asked to sign a form to show that you have given your consent to participate. You are able to change your mind before taking part in this study or during the interview.

What are the benefits and risks of taking part in this study?

There are no promised benefits to taking part in this study. It is hoped that the results will help in improving the quality of care received by women during their pregnancies and the birth of their children.
There are no specific risks or disadvantages to taking part. However, some participants may find it upsetting to talk about their pregnancy and delivery if they had a particularly unpleasant experience. If you feel that this may be the case, please don't feel that it is necessary to take part in the study. If at any point during the interview you wish to stop, this is perfectly acceptable and you will be able to talk to Dr Meza afterwards for further support.
There will be no costs to participate in the study. You will not be paid to take part, however if you are required to attend the clinic on another day you will be reimbursed for travel costs.

Will my information be kept confidential?

If you give your permission to participate in the study, all of your information will be kept confidential. Any paper with your information on will be locked in a filing cabinet and destroyed when it is no longer needed. Electronic copies will be securely stored and deleted when they are no longer needed.
Your name will not be able to be identified as all data belonging to you will be given a unique study number and any names/places will not be used. Only the researchers will have access to your data.

What happens if I don't want to take part in the study anymore?

If you wish to change your mind after you have taken part in the interview, you will be allowed to do so for two days following. This will not affect the care or treatment which you receive at the centre. If you wish to withdraw from the study after your interview, you will find the contact details for the researcher and Dr Meza at the bottom of this participant information sheet.

What happens if I want to complain?

If you have experienced any problems or wish to complain about something, you will be directed to the researcher or Dr Meza who will deal with this.

Further information and contact details

If you wish to take part in this research, after reading the information provided, please speak to your nurse or doctor at the clinic. If you have any further questions or concerns speak to the researcher or Dr Meza.

Contact details:

Harriet Marsland
Medical Student, University of Birmingham

Dr Graciela Meza
Location supervisor, senior academic

Thank you for your interest in the study.
