# Supplementary material for: A qualitative exploration of women’s experiences of antenatal and intrapartum care: The need for a woman-centred approach in the Peruvian Amazon
Source: PLoS One. 2019 Jan 7;14(1):e0209736. doi: 10.1371/journal.pone.0209736 (PMC6322728; doi:10.1371/journal.pone.0209736)
Supplement: S2 Text — (PDF) [file pone.0209736.s002.pdf]

Demographic Questionnaire

STUDY TITLE: Exploring women's experiences and perceptions of Antenatal and Intrapartum Care in the Peruvian Amazon: a qualitative study

Researcher: Harriet Marsland

Participant Study ID Number: ____________

Please answer the following questions, indicating your answer with a tick in the box. Where prompted and appropriate, please provide more written information.

1. What is your age?

   ______ years

2. How long did it take you to travel here today?

   ______ minutes

3. What is your preferred language to communicate in?

   ☐ Spanish ☐ Quechua ☐ English

4. What is your ethnicity?

   ☐ Amerindian ☐ Mestizo ☐ White

   ☐ Black ☐ Japanese ☐ Chinese

   Other (please specify): ______________

5. What is your current marital status?

   ☐ Single ☐ Married

   ☐ Cohabiting/living together ☐ Separated

   ☐ Divorced ☐ Widowed

   ☐ Other (please specify):______________

6. What is the highest level of education you have completed?

   ☐ 11 years (primary school)

   ☐ 16 years (high school)

☐ 18 years

☐ University

Other (please specify): _______________

7. What is your current employment status?

☐ Employed ☐ Unemployed

If you are employed, what do you work as? ______________

8. How many children do you have?

____

9. Your last pregnancy:

    a. Was this your first child?

☐ Yes ☐ No

    b. When did you give birth? ______________
    c. Where did you give birth? ______________
    d. How long was your labour? ______________
