# Supplementary material for: A qualitative exploration of women’s experiences of antenatal and intrapartum care: The need for a woman-centred approach in the Peruvian Amazon
Source: PLoS One. 2019 Jan 7;14(1):e0209736. doi: 10.1371/journal.pone.0209736 (PMC6322728; doi:10.1371/journal.pone.0209736)
Supplement: S3 Text — (PDF) [file pone.0209736.s003.pdf]

<u>Topic Guide</u>

Introduction
I am Harriet Marsland, a student from the University of Birmingham in the United Kingdom.
Thank you for agreeing to participate in this study.

Purpose:
- This study aims to explore women's experiences of care in pregnancy and childbirth in Iquitos.
- It is hoped that it will help to improve the quality of services provided in pregnancy.
- We plan to translate the information gathered from the study into Spanish so that it can be used in Peru for this purpose

Expected duration:
- It is expected that this interview will last between 30 and 60 minutes

Interview content:
- In the interview I will be talking to you about your experiences of antenatal care and birth

Data collection:
- The interview will be recorded using an audio recorder
- What we say will be written on a laptop and used as part of the research

Confidentiality:
- All information gathered will be kept confidential and anonymised
- Your name and other identifiable information will not be present in the written record of the interview.

Questions:
- Do you have any questions?

Consent:
- Please take the consent form and initial/sign in the relevant areas if you are in agreement with the statements.
- I'd just like to remind you that you can withdraw from the study until two days after the interview by contacting Dr Meza using the details provided on the information form
- If you wish to stop this interview at any time please just say.

Background Questionnaire
Can I ask you some background questions?
(Questionnaire to be completed verbally and filled out by researcher)

- I will now turn the audio recorder on, is this still ok?

Topic	Questions & Probes
**Experiences of antenatal care**	Where did you receive your antenatal care?<br><br>Who did you receive your antenatal care from?<br>Did you see the same person each time?<br>How would you describe your relationship with them?<br>Were they male/female?<br>Did you receive care off both genders?<br>How did you feel about this?<br>If both – in which situation did you feel most comfortable?<br>Why do you think this might have been?<br>Would you have liked to have seen anybody else?<br><br>Would you have liked any more or less appointments?<br>Why? When did you have your appointments?<br>At what stage would you have liked to have more appointments?<br><br>How much information were you given about your pregnancy and caring for your child?<br>Who gave this to you and at what stage?<br>Did you receive enough information?<br>No – what more information would you have liked?<br>Who would you have liked to have received this information from?<br>What would have been the best way to receive this information?
**Knowledge of antenatal care**	How important do you think antenatal care is?<br>Why do you think this?<br><br>Do you know what problems might occur without this care?<br>Where did you gain this information?<br><br>What do you believe is involved in care during pregnancy?<br>Do you know what is carried out at each appointment?<br>Do you know the purpose of these tests?

**Barriers, difficulties, motivators or enablers**	Describe what would normally happen on the day of an appointment<br>How do you get to the clinic?<br>How easy is it to get to the clinic for your appointment?<br>Who attends appointments with you?<br><br>What factors made you attend your appointments?<br>What might have stopped you from attending your appointment?<br>Why might this have stopped you?<br>How could you avoid/overcome these factors?<br><br>How was your antenatal care funded?<br>Do you find it difficult to afford the care?<br>Would you be more likely to attend if it was paid for?<br><br>What could be done to make it easier for women to attend antenatal care appointments?<br><br>Who played a part in your decisions about antenatal care?<br>Who gave you information and educated you on your pregnancy?<br>What was your husband's role during the pregnancy?<br><br>How easy is it to get an appointment at the centre?<br>How far in advance can you organize this?<br>Do you get told when you need to come in for your next appointment? How to know to come in for another? Reminders?<br>If not offered, would you find this useful?
**Satisfaction of antenatal care**	What were the best/worst aspects of the antenatal care that you received?<br>Why? What would you have liked to have been done differently?<br><br>If applicable, how does this experience compare to previous pregnancies?<br>Did you have the same number of appointments?<br>Did you feel like you received any less care?<br><br>Do you think that you benefitted from the care that you received during your pregnancy?<br>Why?

**Experience of Intrapartum care**	Describe your experience of the care you received during childbirth.<br>How long were you kept in the healthcare facility for?<br>Were there any complications?<br>How were these dealt with?<br><br>What are your opinions on pain relief in pregnancy?<br>Did you receive any pain relief or medication?<br>Have you used it previously/would you use it again and why?<br>If no – would you have liked to?<br><br>What were your thoughts and feelings during labour?<br>Why did you feel this way?<br><br>Who were the main people supporting/caring for you during labour?<br>Family/friends<br>Staff (what was this relationship like?)<br>How many staff looked after you? Do you know what their roles were?<br><br>What was your opinion of the area in which you gave birth?<br>Did you have any concerns?<br>Did you feel comfortable and relaxed? Why?<br><br>If this was your first pregnancy:<br>Was the pregnancy different to what you had expected?<br>If so how? Why do you think this was?<br><br>Do you think home or healthcare centre births are better?<br>Why do you think this?<br>If you were to have the choice where would you like to give birth?<br>Before this birth, what was your preference?/Has your opinion of this changed at all?

**Satisfaction of Intrapartum Care**	What were the best/worst aspects of your delivery?<br>Why? What would you have liked to have been done differently?<br>How do you think your experience could be improved?<br>Did you feel like you knew what was happening throughout the delivery?<br>Were there any points where you felt you didn't what you should be doing?<br><br>If you have given birth more than once:<br>How does this experience compare to previous pregnancies?<br>In what ways?<br>Why do you think it might have been different?<br>Did you feel like you received any less care/support?<br><br>Do you think that you benefitted from the care that you received during birth?<br>Why?<br><br>Would you recommend this care facility and staff to friends/family?<br>Would you attend this centre again for delivery?
