# Supplementary material for: A qualitative exploration of women’s experiences of antenatal and intrapartum care: The need for a woman-centred approach in the Peruvian Amazon
Source: PLoS One. 2019 Jan 7;14(1):e0209736. doi: 10.1371/journal.pone.0209736 (PMC6322728; doi:10.1371/journal.pone.0209736)
Supplement: S4 Text — (PDF) [file pone.0209736.s004.pdf]

Basic Outline of Antenatal Care

1st appointment	<14 weeks
2nd appointment	14-21 weeks
3rd appointment	22-24 weeks
4th appointment	25-32 weeks
5th appointment	33-36 weeks
6th appointment	37-40 weeks

This is the basic outline of antenatal care as recommended and documented by the Ministerio de Salud Peru, December 2013.
