# Supplementary material for: A qualitative exploration of women’s experiences of antenatal and intrapartum care: The need for a woman-centred approach in the Peruvian Amazon
Source: PLoS One. 2019 Jan 7;14(1):e0209736. doi: 10.1371/journal.pone.0209736 (PMC6322728; doi:10.1371/journal.pone.0209736)
Supplement: S1 Checklist — (PDF) [file pone.0209736.s005.pdf]

**Manuscript:** A qualitative exploration of women's experiences of antenatal and intrapartum care: the need for a woman-centred approach in the Peruvian Amazon.

Harriet Marsland, Graciela Meza, Gilles de Wildt, Laura L. Jones.

# Consolidated criteria for reporting qualitative studies (COREQ): 32-item checklist

Developed from:
Tong A, Sainsbury P, Craig J. Consolidated criteria for reporting qualitative research (COREQ): a 32-item checklist for interviews and focus groups. *International Journal for Quality in Health Care*. 2007. Volume 19, Number 6: pp. 349 – 357

| No. Item | Guide questions/description | Reported on Page # |
|---|---|---|
| **Domain 1: Research team and reflexivity** | | |
| *Personal Characteristics* | | |
| 1. Inter viewer/facilitator | Which author/s conducted the inter view or focus group? | Harriet Marsland |
| 2. Credentials | What were the researcher's credentials? E.g. PhD, MD | Harriet Marsland, BMedSc (Hons)<br><br>Dr Gilles de Wildt, MRCGP, MSc<br><br>Dr Graciela Meza, MC, MSP<br><br>Dr Laura Jones, BSc (Hons), PhD |
| 3. Occupation | What was their occupation at the time of the study? | Harriet Marsland, International Health/Medical Student, University of Birmingham<br><br>Dr Gilles de Wildt, General practitioner, International Health BMedSci Course Lead University of Birmingham |

| | | |
|---|---|---|
| | | Dr Graciela Meza, Full Time Assistant Professor, Faculdad Medicinade, Iquitos, General Practitioner<br><br>Dr Laura Jones, Lecturer in Qualitative and Mixed-Methods Applied Health Research, University of Birmingham |
| 4. Gender | Was the researcher male or female? | Female |
| 5. Experience and training | What experience or training did the researcher have? | Methods – the researcher had previously carried out a small scale practice qualitative study as part of her intercalated degree at University of Birmingham. |
| *Relationship with participants* | | |
| 6. Relationship established | Was a relationship established prior to study commencement? | No<br>. |
| 7. Participant knowledge of the interviewer | What did the participants know about the researcher? e.g. personal goals, reasons for doing the research | Information provided on Participant Information Sheet and prior to each interview (see S1 and S3) |
| 8. Interviewer characteristics | What characteristics were reported about the inter viewer/facilitator? e.g. Bias, assumptions, reasons and interests in the research topic | Participant Information Sheet S1 and Methods |

| | | |
|---|---|---|
| **Domain 2: study design** | | |
| *Theoretical framework* | | |
| 9. Methodological orientation and Theory | What methodological orientation was stated to underpin the study? e.g. grounded theory, discourse analysis, ethnography, phenomenology, content analysis | Methods |
| *Participant selection* | | |
| 10. Sampling | How were participants selected? e.g. purposive, convenience, consecutive, snowball | Methods |
| 11. Method of approach | How were participants approached? e.g. face-to-face, telephone, mail, email | Methods |
| 12. Sample size | How many participants were in the study? | Methods |
| 13. Non-participation | How many people refused to participate or dropped out? Reasons? | No participants dropped out |
| *Setting* | | |
| 14. Setting of data collection | Where was the data collected? e.g. home, clinic, workplace | Methods . |
| 15. Presence of non-participants | Was anyone else present besides the participants and researchers? | Methods |
| 16. Description of sample | What are the important characteristics of the sample? e.g. demographic data, date | Table 1 |
| *Data collection* | | |
| 17. Interview guide | Were questions, prompts, guides provided by the authors? Was it pilot tested? | See S3 |
| 18. Repeat interviews | Were repeat inter views carried out? If yes, how many? | No |
| 19. Audio/visual recording | Did the research use audio or visual recording to collect the data? | Methods |

| | | |
|---|---|---|
| 20. Field notes | Were field notes made during and/or after the inter view or focus group? | No |
| 21. Duration | What was the duration of the inter views or focus group? | Results |
| 22. Data saturation | Was data saturation discussed? | Methods |
| 23. Transcripts returned | Were transcripts returned to participants for comment and/or correction? | No |
| **Domain 3: analysis and findings** | | |
| *Data analysis* | | |
| 24. Number of data coders | How many data coders coded the data? | Methods |
| 25. Description of the coding tree | Did authors provide a description of the coding tree? | No |
| 26. Derivation of themes | Were themes identified in advance or derived from the data? | Methods |
| 27. Software | What software, if applicable, was used to manage the data? | None |
| 28. Participant checking | Did participants provide feedback on the findings? | No |
| *Reporting* | | |
| 29. Quotations presented | Were participant quotations presented to illustrate the themes/findings? Was each quotation identified? e.g. participant number | Embedded throughout results section |
| 30. Data and findings consistent | Was there consistency between the data presented and the findings? | Results and Discussion |
| 31. Clarity of major themes | Were major themes clearly presented in the findings? | Results |
| 32. Clarity of minor themes | Is there a description of diverse cases or discussion of minor themes? | Results |
